# Virtual Preoperative Education in Bariatric Surgery: Patient Acceptance and Travel-Related Carbon Savings

**DOI:** 10.1007/s11695-026-08840-w

**Published:** 2026-07-17

**Authors:** Justus Schmid, Koroush Kabir, Alexander Bäuerle, Esther Maria Bonrath, Barbara König, Till Hasenberg

**Affiliations:** 1 Helios Obesity Center West, Helios St. Elisabeth Hospital Oberhausen, Oberhausen, NRW Germany; 2 Department of Orthopedics and Trauma Surgery, Helios University Hospital, University Witten/Herdecke, Wuppertal, Germany; 3 Witten/Herdecke University, Witten, Germany; 4 Clinic for Psychosomatic Medicine and Psychotherapy, LVR-University Hospital Essen, University of Duisburg-Essen, Essen, Wuppertal, Germany; 5 Center for Translational Neuro- and Behavioral Sciences (C-TNBS), University of Duisburg-Essen, Essen, Germany; 6 Clinic for Surgery & Hand Surgery, Regensburg, Germany; 7 Sana Obesity Center North Rhine-Westphalia, Remscheid/Düsseldorf, Germany

**Keywords:** Weight loss surgery, Digital health services, Online patient education, CO_2_ reduction, Patient mobility, Healthcare sustainability, Pre-operative management

## Abstract

**Background:**

Comprehensive preoperative patient education is critical for optimizing postoperative outcomes and long-term weight management after bariatric surgery. Traditional in-person educational sessions, however, generate considerable greenhouse gas emissions, primarily due to participant travel. Telemedicine offers an environmentally sustainable alternative with the potential to reduce the carbon footprint within obesity care. This study aimed to quantify savings in greenhouse gas emissions and assess patient acceptance of virtual preoperative obesity educational sessions compared to conventional in-person courses.

**Methods:**

Between January and December 2024, *N* = 183 patients attending a virtual preoperative educational seminar for individuals with obesity—integrated into standard bariatric surgical preparation—were surveyed regarding their hypothetical travel behavior, had the seminar been held in person. Distances from participants’ residences to the seminar location and preferred transportation modes were used to calculate avoided CO₂ emissions utilizing the Transport Emission Model (TREMOD) developed by the German Federal Environment Agency.

**Results:**

The average one-way travel distance was 33.08 km, with 83.6% of participants indicating they would have traveled by private vehicle, predominantly powered by internal combustion engines. Transitioning to the virtual format resulted in a total avoidance of approximately 1.792 metric tons of CO₂ equivalent emissions over the study period. Patient acceptance of the virtual format was high, with 93.4% of the cohort rating it as equivalent or superior to in-person sessions. Notably, 25% of the participants stated they would not have attended the mandatory session if held exclusively in person, indicating that the virtual format successfully bridged a significant attendance gap.

**Conclusions:**

Given the increasing prevalence of obesity and the critical role of preoperative education in bariatric care, telemedicine represents an effective strategy to meaningfully reduce travel-related emissions. Virtual educational interventions eliminate CO₂-intensive journeys while maintaining broad accessibility and high patient satisfaction. Integrating telemedicine into obesity surgery care pathways can thus significantly contribute to sustainable, climate-conscious healthcare delivery.

## Introduction

 Obesity remains a complex, systemic disease, driving significant comorbid risks such as type 2 diabetes mellitus, cardiovascular disease, certain cancers, and metabolic syndrome [1, 2]. As prevalence rises worldwide, obesity imposes a heavy burden on individual health, quality of life, and healthcare resources. Effective management requires appropriate patient education and information on topics such as nutrition, physical activity, and preparation for bariatric surgery, as well as lifelong follow-up [1]. However, in-person seminars and consultations force individuals with obesity to travel, often long distances depending on the care setting, creating logistical barriers and contributing to healthcare’s carbon footprint.

Globally, the healthcare sector accounts for approximately 4.4% of net greenhouse gas emissions—if viewed as a country, it would rank fifth in emissions output [3]. In the United States, healthcare contributes roughly 8.5% of national CO₂ emissions (equivalent to the 11th largest emitter worldwide) [4], and in the United Kingdom, the National Health Service is responsible for about 4–5% of the nation’s total carbon footprint [5]. In Germany, healthcare alone generates 0.5–1 ton of CO₂ per capita, with transportation accounting for about 7% of these emissions [3]. Consequently, reducing travel-related emissions in obesity care is both an environmental and a public health priority. Beyond environmental concerns, conventional on-site attendance presents formidable obstacles for patients with severe obesity; mobility impairments and chronic joint pain often preclude travel to centralized surgical centers. Reducing the travel burden is therefore not only an ecological priority but also a clinical necessity to ensure patient participation and preoperative readiness.

Telemedicine offers a viable alternative by enabling patient education and clinical consultations to occur entirely online [6, 7]. By eliminating the need for travel, virtual formats directly reduce CO₂ emissions associated with transport. Multiple studies in oncology, urology, bariatrics and orthopedics have demonstrated significant CO₂ savings when shifting from face-to-face interactions to telehealth [8–12]. In the context of obesity, virtual preoperative seminars can deliver the same comprehensive content—covering disease pathophysiology, lifestyle modifications, and bariatric surgery expectations—while sparing patients the environmental and practical costs of physical attendance [13].

This study aimed to quantify the reduction in greenhouse gas emissions resulting from the conversion of a monthly, mandatory preoperative bariatric patient education session from an in-person format to a virtual platform (implemented since the COVID-19 pandemic). By comparing the emissions associated with participants’ travel to the seminar location with the carbon footprint of conducting the session online, the study seeks to evaluate the potential contribution of telemedicine to CO₂ emission savings in obesity care.

## Methods

This study exclusively involved participants of a virtual patient education session designed for individuals living with obesity, conducted between January and December 2024. This study was based on a single mandatory attendance at a 60-minute seminar, which represents an integral component of the standardized preoperative pathway for bariatric surgery candidates. The final sample consisted of *N* = 183 participants, aged between 18 and 71 years (M = 41.3, SD = 10.8).

Following the educational session, participants were invited to complete an online survey via a dedicated link. Prior to participation, all individuals received comprehensive information about the study’s purpose, procedures, data confidentiality, and voluntary nature of the survey. Informed consent was obtained electronically before accessing the online survey. No financial compensation was offered. The questionnaire was divided into three sections: the first evaluated participants’ satisfaction with and acceptance of the virtual seminar; the second assessed anticipated travel behavior in the context of a hypothetical in-person seminar; and the third collected sociodemographic information, including postal code, age, gender, height, and weight.

## Data Analysis

Data analysis was performed using Microsoft Excel for Microsoft 365 (Version 2504). Initially, the driving route in kilometers between the hypothetical seminar venue and the participants’ postal code areas was calculated. For this purpose, a tool from the website “Suche-Postleitzahl.org” (Schwochow Softwareentwicklung, Vollmersbach, Germany) was used. Potentially avoided greenhouse gas emissions were estimated using the Transport Emission Model (TREMOD), developed by the German Federal Environment Agency [14]. The calculation of pollutant emissions from road traffic is based on emission factors provided in the Emission Factors Handbook (HBEFA) [14] (see Table 1). To distinguish between local and long-distance traffic emissions, average values were computed accordingly [15]. Data on participant satisfaction and acceptance were analyzed descriptively to assess the perceived quality of the virtual format compared to conventional in-person consultations. This allows for a comprehensive evaluation of telemedicine not only as an ecological tool but also as a clinically accepted educational modality.

**Table 1:** Comparison of the average emissions of various modes of transport in passenger traffic in Germany [14]

Transportation means	Greenhouse gases ^1^	Nitrogen oxides	Particulate matter	Utilization
Private car	166	0.32	0.013	1.4 Pers./car
Of which, electric car	79	0.08	0.004	
Of which, hybrid car	121	0.10	0.008	
Of which, diesel	173	0.52	0.015	
Of which, petrol	165	0.17	0.012	
Average long distance traffic	**31.00**	**0.04**	**0.002**	**49%**
Average short distance traffic	**71.33**	**0.16**	**0.0053**	**18.3%**
Motorcycle	**151.29**	**0.16**	**0.015**	**1 Pers./motorcycle**

g/Pkm = grams per person-kilometer, including emissions from the provision and conversion of energy carriers into electricity, gasoline, diesel, liquefied natural gas, and natural gas

^1^ CO_2_, CH_4_ and N_2_O, expressed in CO_2_ equivalents according to AR5 (5th Assessment Report of the IPCC)

## Results

Among the *N* = 183 participants preparing for bariatric surgery, the gender distribution was 83.6% female and 16.4% male. The mean Body Mass Index (BMI) was 47.3 kg/m², ranging from 30.9 to 69.7 kg/m², reflecting a population with severe obesity.

**Fig. 1:**
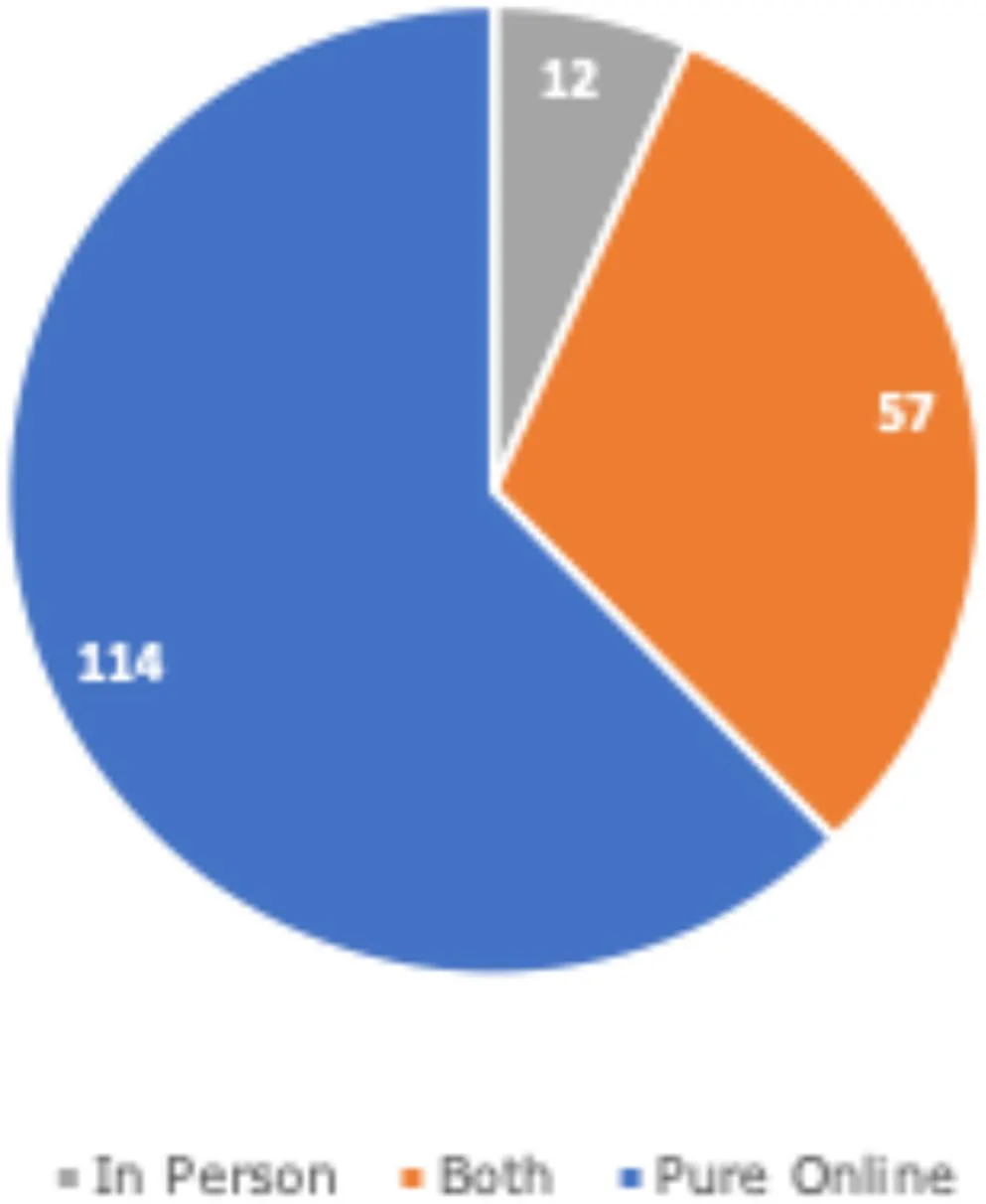
Preferred format of the virtual preoperative education seminar for individuals living with obesity (*n* = 183)

Nearly three-quarters of respondents (*n* = 129) indicated they would have attended the patient education session in person, which is a mandatory component of preoperative preparation for bariatric surgery, while just over one-quarter (*n* = 54) would have participated exclusively online. When offered a choice of format, 6.6% (*n* = 12) preferred an exclusively in-person event, 31.1% considered both in-person and online participation equally acceptable, and 62.3% favored purely online attendance (see Fig. 1). Notably, 46 patients (25% of the cohort) reported they would not have attended the mandatory session at all had it been offered in person only, indicating that the virtual format bridged a relevant attendance gap and ensured that these patients received critical preoperative information they would otherwise have missed.

While the carbon footprint analysis included the entire cohort (*N* = 183), a more nuanced analysis of educational quality was conducted among a subgroup of 102 participants (55.7%) who had already attended an in-person consultation. This subgroup analysis provides a direct comparison between the two modalities based on first-hand patient experience. These consultations, led by one of two senior physicians (T.H. or B.K.), covered the same core topics included in the online seminar, providing a basis for direct comparison between the modalities.

The results reveal a clear preference for the online format or a neutral assessment across all five thematic areas related to obesity surgery.

On average, 40.6% of respondents rated the online explanations as better, while only 15.9% preferred the in-person consultation. A substantial proportion of 43.5% found the explanations equally effective in both formats (“neutral”).

A detailed breakdown of preferences by thematic area is presented in Table 2.

**Table 2:** Participant preferences for online vs. In-person explanation by thematic area (N = 102): n = number of participants % = percentage of respondents in each category

Thematic Area	Online Format Preferred (*n*/%)	In-Person Format Preferred (*n*/%)	No Preference/Equal Rating (*n*/%)
What is Obesity?	45 (44.1%)	16 (15.7%)	41 (40.2%)
Why does Obesity Occur?	44 (43.1%)	14 (13.7%)	44 (43.1%)
Why must Obesity be Treated?	38 (37.2%)	11 (10.8%)	53 (52.0%)
Which Therapy Options are Available for Obesity Treatment?	31 (30.4%)	28 (27.4%)	43 (42.2%)
Complications in Bariatric Surgery	49 (48.0%)	12 (11.8%)	41 (40.2%)
Average	**41 (40.6%)**	**16 (15.9%)**	**44 (43.5%)**

The average distance between participants’ residences and the in-person seminar site was approximately 33.08 km (round-trip distance: 66.16 km).

**Fig. 2:**
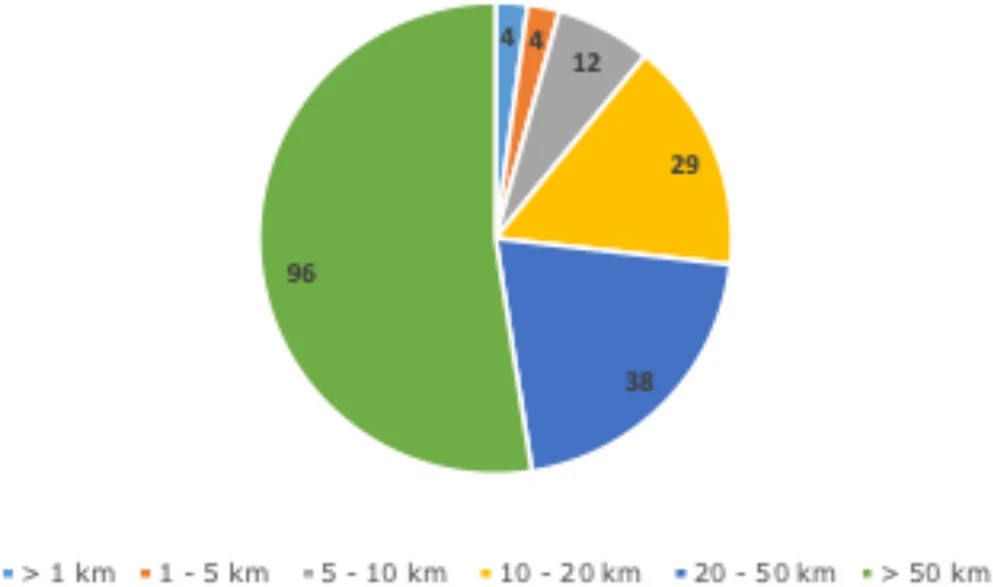
Theoretical total travel distance to the in-person preoperative education seminar for individuals with obesity (*n* = 183)

The distribution of individual total travel distances was as follows: 2.2% would have traveled less than 1 km, 2.2% between 1 and 5 km, 6.6% between 5 and 10 km, 15.8% between 10 and 20 km, 20.8% between 20 and 50 km, and 52.5% over 50 km (see Fig. 2).

**Fig. 3:**
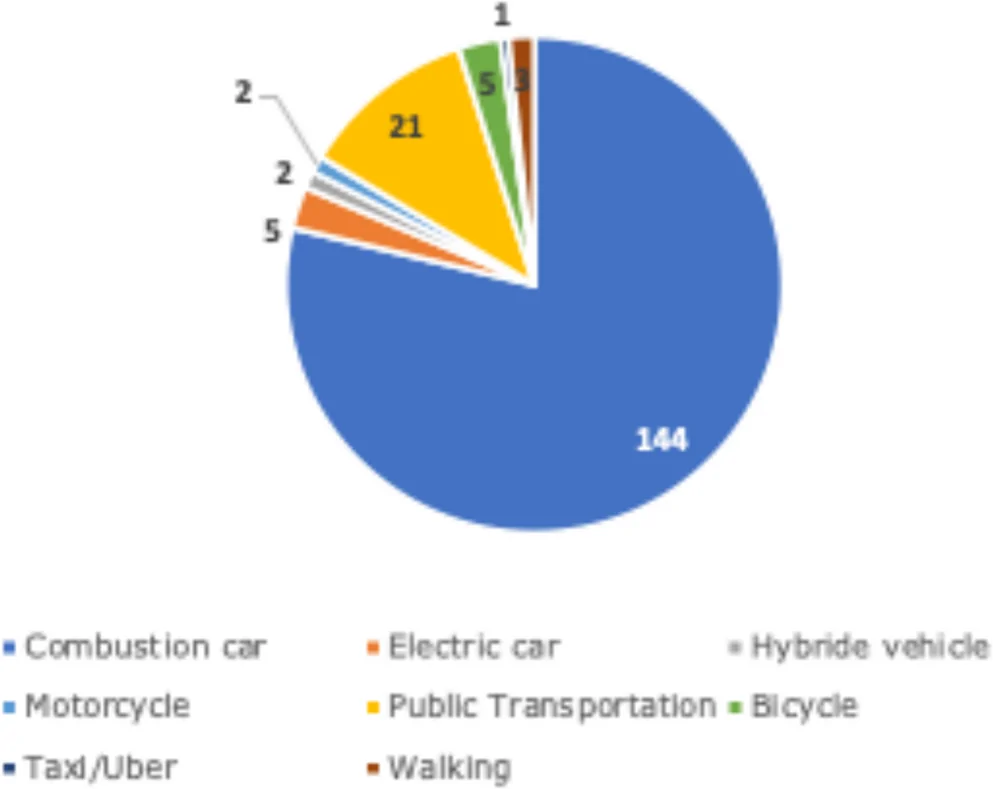
Theoretical mode of transport to the in-person preoperative education seminar for individuals with obesity (*n* = 183)

In terms of transportation, 83.6% would have used a private vehicle (combustion engine car: *n* = 144, electric car: *n* = 5, hybrid vehicle: *n* = 2) or motorcycle (*n* = 2). Public transportation was utilized by 11.5%, with one-third opting for long distance and two-thirds for local transit. Additionally, 2.7% would have cycled, 0.6% used taxi or ridesharing, and 1.6% traveled on foot (see Fig. 3).

The estimated total distance of 12,107 km, combined with the modes of transport used, would have resulted in emissions of approximately 1.792 tons of CO₂ equivalents, 1.914 kg of nitrogen oxides (NOx), and 0.13 kg of particulate matter.

## Discussion

This study demonstrates that converting an in-person patient education seminar for individuals living with obesity into a fully virtual format can meaningfully reduce travel-related greenhouse gas emissions. The implications of this finding extend beyond mere environmental considerations; they also touch on accessibility, patient adherence, and the evolving landscape of bariatric care. In bariatric surgery—where comprehensive preoperative education is critical to optimizing surgical outcomes, ensuring patient safety, and promoting long-term weight loss maintenance—telemedicine emerges as a multidimensional solution that addresses clinical, logistical, and ecological challenges in tandem.

The environmental advantages of telemedicine have been documented across multiple healthcare domains. In oncology, for example, a Welsh study investigated the carbon footprint of multidisciplinary tumor board meetings. By replacing face-to-face meetings with videoconference sessions, teams reduced their annual travel distance by 18,000 to 20,800 km, translating to CO₂ emission reductions between approximately 1.7 and 2.6 tons [11]. In the United Kingdom, the 2019 introduction of virtual follow-up clinics in urology resulted in annual savings between 1.04 and 4.04 tons of CO₂, attributable to avoided patient and provider travel [10]. In Spain, efforts to shift 640,122 outpatient appointments online in 2022 prevented nearly 1,957 tons of CO₂ emissions [16]. Similarly, a study at the Department of Orthopedics and Trauma Surgery at the University Hospital, Bonn, Germany revealed that shifting outpatient visits to video consultations during the COVID-19 pandemic reduced transport-related emissions by up to 97% [9].

Within the field of bariatrics, the potential for carbon savings is especially pronounced. A 2023 retrospective U.S. analysis found that when preoperative consultations and educational sessions for bariatric surgery patients transitioned to a telemedicine platform, greenhouse gas emissions decreased by approximately 80% [8]. This is partly because bariatric candidates often travel long distances to access specialized centers—due to the centralization of obesity surgery services and the need for multidisciplinary evaluation by surgeons, dietitians and psychologists. These benefits were also confirmed in a recent study by Conte et al. [12], which analyzed 338 bariatric surgery patients who underwent remote preoperative assessments via Zoom. The study reported a 99.97% reduction in CO₂ emissions from avoided travel, with no changes to surgical indication or perioperative management. Complication rates and outcomes were comparable to traditional care, underlining that remote formats can preserve clinical safety while dramatically improving environmental sustainability [12].

A 2020 systematic review by Purohit et al. further emphasizes the ecological advantage of telemedicine in healthcare, particularly regarding transport-related emissions. By synthesizing data from diverse studies—ranging from cardiology to primary care—Purohit et al. concluded that telemedicine interventions generally reduce carbon emissions whenever patients would otherwise need to travel more than a few kilometers [17]. Holmner et al. quantified this threshold, demonstrating that once round-trip distances exceed approximately 7.2 km, the emissions from travel outweigh those generated by telemedicine technologies (including data centers, network infrastructure, and end-user devices) [18]. Given that bariatric seminars at tertiary centers often involve one-way distances of 30 km or more (as in our study, where the mean travel distance was 33.08 km), telemedicine becomes environmentally advantageous in nearly all bariatric preoperative contexts.

Patient education seminars prior to bariatric procedures typically cover the pathophysiology of obesity, nutritional guidelines, behavioral modification strategies, postoperative expectations (e.g., dietary progression, exercise, supplement use), and potential risks and complications. This comprehensive education is essential for minimizing perioperative morbidity, reducing postoperative complications, and increasing long-term adherence to lifestyle changes [13].

However, the logistics of attending in-person seminars pose significant challenges. Many bariatric centers are geographically centralized, requiring patients—often individuals with mobility limitations associated with obesity—to travel lengthy distances, sometimes exceeding an hour each way. In addition to the burden of arranging transportation, patients may face time off work, childcare coordination, and out-of-pocket travel expenses [19]. These barriers can contribute to appointment no-shows, delayed surgery scheduling, and ultimately suboptimal preoperative preparation. Moreover, when patients fail to attend essential education sessions, they may lack understanding of postoperative requirements, increasing the risk of nutritional deficiencies, weight regain, or surgical complications.

By providing virtual preoperative education, telemedicine resolves logistical challenges, enhances accessibility (especially for remote patients), and lowers the environmental footprint. It maintains educational quality and interactivity through various online tools.

Several studies have documented high patient satisfaction with virtual medical preoperative education. In our cohort, 93.4% of participants indicated they preferred or found online participation equivalent to face-to-face seminars. Beyond the quantifiable environmental benefits, our study also reveals a strong qualitative endorsement of virtual education from the patient perspective. The detailed analysis of participant satisfaction, presented in Table 2, demonstrates a high level of acceptance for the online format. Notably, complex and sensitive topics such as ‘Complications in Bariatric Surgery’ garnered a significant preference for online explanations (48.0% preferred the online format), suggesting that digital platforms effectively convey intricate medical content. This preference may stem from the ability to review information at one’s own pace, the reduced social anxiety associated with in-person discussions of sensitive health issues, or the perceived privacy of the virtual setting. Furthermore, the consistently high proportion of neutral responses across all topics (average 43.5%) indicates that for a substantial number of patients, the online format is considered at least equivalent to traditional in-person consultations in terms of informational quality and clarity. These findings collectively reinforce that telemedicine-based education is not merely an environmentally sustainable alternative, but a high-quality, patient-centered approach that effectively lowers access barriers, enhances reach, and promotes inclusivity in bariatric care. Digital delivery may offer a ‘safe space’ that reduces social anxiety, allowing patients to process risk-related information more effectively than in traditional settings. These findings suggest that virtual education may be perceived as at least equivalent to in-person education for key informational domains, although objective measures of knowledge retention were not assessed.

Comparable satisfaction rates have been reported elsewhere: a U.S. “Minute Clinic” telemedicine study found > 94% satisfaction, with one-third of participants preferring remote visits over in-person consultations [20]. In the UK, a virtual urology clinic achieved > 90% positive feedback, and orthopedic telehealth consultations at Bonn University Hospital (Germany) garnered 84% patient satisfaction [9, 10]. Similar results were also found in cancer care, where a German survey showed that 84% of patients had high or moderate acceptance of video consultations, influenced by factors like younger age, female gender, digital confidence, and positive perceptions of usability and support [21]. This highlights that patients value personalized, accessible digital healthcare for its convenience and flexibility without compromising educational quality or personal connection.

Telemedicine promotes the standardized delivery of educational content by ensuring that all participants receive the same information simultaneously when seminars are delivered live via secure videoconference, thus reducing variability seen in repeated small in-person groups. Additionally, recorded sessions combined with live interaction offer further reinforcement, allowing patients to replay modules at their convenience to review critical points (e.g., post-bariatric dietary progression). Furthermore, telemedicine facilitates data collection through integrated electronic surveys and quizzes, enabling educators to assess patient comprehension and tailor follow-up discussions to address knowledge gaps. This approach has been shown to enhance the effectiveness of digital health interventions by providing personalized education and improving patient engagement [22].

In our study, participants’ average round-trip travel distance to the in-person seminar was 66.16 km. Assuming 83.6% used personal vehicles and 11.5% public transportation, the total travel distance of 12,107 km would have generated approximately 1.792 tons of CO₂ equivalent, 1.914 kg of nitrogen oxides, and 0.13 kg of particulate matter. Holding the seminar virtually effectively averted these emissions. According to Holmner et al. (2014), a 60-minute video consultation—including energy use from servers, devices, and data transmission—generates only about 0.02 kg of CO₂ equivalent emissions per participant [18]. In our study, a single 60-minute virtual session with 183 participants saved approximately 1.792 metric tons of CO₂ equivalent emissions compared to in-person attendance, mainly by avoiding travel. These findings are consistent with Conte et al. (2025), who evaluated a remote bariatric care model and found a reduction of over 50 metric tons of CO₂ equivalent emissions, achieved solely by eliminating patient travel. Their study underscores the broader ecological potential of telemedicine in high-volume surgical pathways like bariatrics.

These findings align with Lenzen et al. [23], who showed that health care contributes between 1% and 5% of global environmental impacts, including greenhouse gas emissions, air pollutants, and water use, which in turn pose risks to human health [23]. Patient travel is a major contributor to this footprint. Consequently, expanding virtual services in bariatric care, including preoperative counseling, nutritional education, and follow-up appointments could significantly reduce the sector’s environmental impact. Overall, these results highlight the considerable potential of telemedicine in obesity and bariatric care to lower healthcare’s carbon footprint through scalable digital solutions.

High acceptance of telemedicine is particularly relevant among individuals seeking bariatric treatment, as both physical limitations (such as joint pain and reduced mobility) and psychological factors (including obesity-related stigma, social anxiety, and depressive symptoms) may act as significant barriers to in-person participation. In our cohort, 25% of participants indicated they would not have attended the in-person seminar, underscoring potential attendance gaps in traditional models. By offering virtual education, bariatric surgery programs can ensure that these individuals still receive critical information, thus enhancing equity of access.

However, equitable access to telemedicine requires addressing potential digital divides. In our study, all participants had internet access and basic digital literacy. However, the ‘digital divide’ remains a critical barrier in broader populations. Lower socioeconomic status or limited digital literacy could lead to the exclusion of certain patient groups from virtual care [24]. Future bariatric care models should therefore consider hybrid approaches to ensure that no group is disadvantaged by the transition to digital formats. This is consistent with findings from other chronic disease populations. For example, studies in patients with diabetes and chronic pain have demonstrated that acceptance of eHealth or e-mental health interventions tends to be moderate, and that core predictors from the Unified Theory of Acceptance and Use of Technology (UTAUT)—namely performance expectancy, effort expectancy, and social influence—play a key role in shaping patient attitudes toward digital tools. In both populations, extended UTAUT models (including mental health and sociodemographic variables) explained a significant proportion of variance in acceptance, highlighting the need to tailor digital interventions to individual needs and capabilities [25, 26]. Importantly, recent research specifically targeting patients with obesity confirms the relevance of these findings for bariatric care. A recent evaluation of a structured educational program for patients awaiting bariatric surgery demonstrated significant reductions in anxiety, depression, and stress symptoms, with patients reporting high perceived benefit. Notably, outcomes were comparable between face-to-face and videoconferencing formats—supporting virtual delivery as an effective and acceptable option [13]. In a broader context, digital interventions in obesity care require tailored implementation strategies that address both technological access and individual psychological readiness, as highlighted by UTAUT-based models explaining up to 73.6% of variance in user acceptance [27].

### Limitations and Future Directions

While our study demonstrates notable emission savings from virtual seminars, several limitations must be acknowledged. Because travel behavior was assessed hypothetically rather than observed, actual emissions avoided may differ from our estimates. However, the use of participants’ postal codes, stated transport preferences, and established TREMOD emission factors provides a transparent and reproducible approximation of travel-related emissions. In addition, emissions from virtual sessions were estimated indirectly; future studies should include real-time data on energy use and digital infrastructure. Moreover, our analysis focused exclusively on preoperative education, leaving the environmental impact of a fully digital bariatric care model including tele-nutrition, psychological support, and follow-up yet to be explored.

Satisfaction data were collected immediately after the online seminar. Due to the anonymous nature of the survey, we were unable to determine when each participant had attended their in-person consultation. The anonymity of the survey prevents us from controlling for recall bias regarding the time elapsed between the in-person consultation and the virtual seminar. Future prospective, randomized trials are necessary to objectively compare knowledge retention and the psychological impact of both modalities in real time. On a broader scale, expanding telemedicine could support healthcare decarbonization goals. Policymakers should promote digital infrastructure, create reimbursement models, and include environmental metrics in bariatric quality programs.

## Conclusion

In conclusion, virtual preoperative education in bariatric surgery represents a ‘triple-win’ strategy: it significantly reduces the healthcare carbon footprint, improves equity of access for patients with mobility limitations, and was perceived by patients as providing high-quality clinical information delivery. Integrating these digital pathways is an essential step toward a modern, patient-centered, and sustainable surgical practice.

## Data Availability

No datasets were generated or analysed during the current study.
